# Crystal structure of 2-{[(2-chloro­phen­yl)imino]­meth­yl}phenol

**DOI:** 10.1107/S2056989014026978

**Published:** 2015-01-01

**Authors:** Matheswaran Saranya, Annamalai Subashini, Chidambaram Arunagiri, Packianathan Thomas Muthiah

**Affiliations:** a PG & Research Department of Chemistry, Seethalakshmi Ramaswami College, Tiruchirappalli 620 002, Tamil Nadu, India; b PG & Research Department of Physics, Government Arts College, Ariyalur 621 713, Tamil Nadu, India; c School of Chemistry, Bharathidasan University, Tiruchirappalli 620 024, Tamil Nadu, India

**Keywords:** crystal structure, 2-{[(2-chloro­phen­yl)imino]­meth­yl}phenol, Schiff base, van der Waals contacts

## Abstract

In the title compound, C_13_H_10_ClNO, the dihedral angle between the planes of the aromatic rings is 51.42 (9)° and an intra­molecular O—H⋯N hydrogen bond closes an *S*(6) ring. The Cl atom and the N atom are *syn*. No directional inter­actions beyond van der Waals contacts are observed in the crystal.

## Related literature   

For related structures recently reported by us and background to Schiff bases, see: Arunagiri *et al.* (2013*a* ▸,*b* ▸). For a related structure, see: Chumakov *et al.* (2005 ▸). 
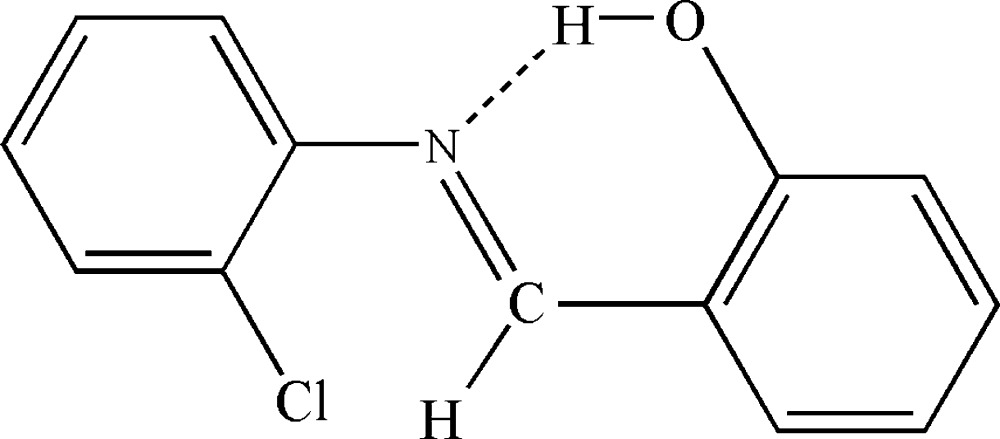


## Experimental   

### Crystal data  

C_13_H_10_ClNO
*M* *_r_* = 231.67
Orthorhombic, 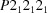

*a* = 6.8591 (2) Å
*b* = 12.1829 (4) Å
*c* = 13.5405 (5) Å
*V* = 1131.50 (6) Å^3^
*Z* = 4
Mo *K*α radiation
μ = 0.31 mm^−1^
*T* = 293 K
0.30 × 0.25 × 0.20 mm

### Data collection  

Bruker Kappa APEXII CCD diffractometer
6509 measured reflections
2744 independent reflections
2315 reflections with *I* > 2σ(*I*)
*R*_int_ = 0.017

### Refinement  

*R*[*F* ^2^ > 2σ(*F* ^2^)] = 0.034
*wR*(*F* ^2^) = 0.097
*S* = 1.06
2744 reflections
146 parameters
H-atom parameters constrained
Δρ_max_ = 0.17 e Å^−3^
Δρ_min_ = −0.17 e Å^−3^
Absolute structure: Flack (1983 ▸)
Absolute structure parameter: 0.01 (7)

### 

Data collection: *APEX2* (Bruker, 2008 ▸); cell refinement: *SAINT* (Bruker, 2008 ▸); data reduction: *SAINT*; program(s) used to solve structure: *SHELXS97* (Sheldrick, 2008 ▸); program(s) used to refine structure: *SHELXL97* (Sheldrick, 2008 ▸); molecular graphics: *PLATON* (Spek, 2009 ▸); software used to prepare material for publication: *PLATON*.

## Supplementary Material

Crystal structure: contains datablock(s) global, I. DOI: 10.1107/S2056989014026978/hb7335sup1.cif

Structure factors: contains datablock(s) I. DOI: 10.1107/S2056989014026978/hb7335Isup2.hkl

Supporting information file. DOI: 10.1107/S2056989014026978/hb7335Isup3.cml

**Fig. 1.**
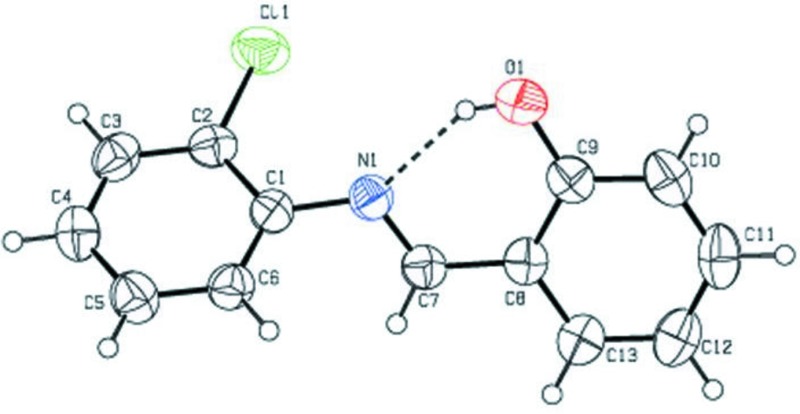
Molecular structure of the title compound with displacement ellipsoids drawn at 50% probability level. Dashed line indicates intramolecular hydrogen bond.

. DOI: 10.1107/S2056989014026978/hb7335fig1.tif
Mol­ecular structure of the title compound with displacement ellipsoids drawn at 50% probability level. Dashed line indicates intra­molecular hydrogen bond.

**Fig. 2.**
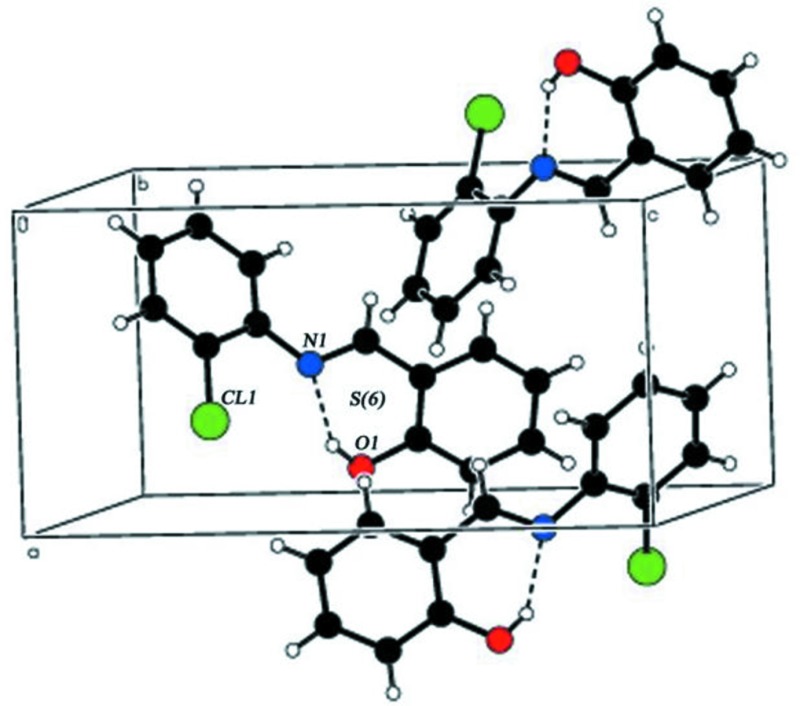
Hydrogen bonding interaction of title compound.

. DOI: 10.1107/S2056989014026978/hb7335fig2.tif
Hydrogen bonding inter­action of title compound.

CCDC reference: 1038374

Additional supporting information:  crystallographic information; 3D view; checkCIF report

## Figures and Tables

**Table 1:** Hydrogen-bond geometry (, )

*D*H*A*	*D*H	H*A*	*D* *A*	*D*H*A*
O1H1N1	0.82	1.88	2.611(2)	147

## supplementary crystallographic information

### S1. Comment

As part of our ongoing studies of Schiff bases (Arunagiri *et al.*, 2013a,b), we now describe the synthesis and structure of the title compound.

An *ORTEP* view of the asymmetric unit is shown in Figure 1. The asymmetric unit contains a molecule of Schiff base. The compound crystallizes in the orthorhombic space group *P*2_1_2_1_2_1_. The dihedral angle between the salicylidene moiety and amino phenyl plane is 51.42 (9)°. The two torsional angles τ1 (N—C—C—C) and τ2 (C—N—C—C) defining the confirmation of the molecule. In the present crystal structure, the torsion angles are 3.2 (3)° (N1—C7—C8—C9), -179.23 (2)° (N1—C7—C8—C13), 47.5 (2)° (C7—N1—C1—C6), -174.48 (2)° (C8—C7—N1—C1) and -135.60 (2)° (N1—C7—C1—C2). The N1—C7 distance of 1.275 (2) Å is normal double bond values and agree well with those observed in other azomethines. The C1—N1—C7 bond angle of 118.70 (2)° in the Schiff base ligand has a normal value. The C3—C2—C1 angle is 121.15 (2)° is larger than typical hexagonal of 120°. The C8—C9—C10 angle is 119.53 (2)° is smaller than typical hexagonal of 120°. This is due to effect of substitution on Cl & OH of the two aromatic rings. The two benzene rings (amino phenyl and salicylaldehyde) and the azomethine group are practically coplanar, as a result of intramolecular O—H···N (O1—H1···N1 with bond length of 2.611 (2) Å and bond angle of 147°) hydrogen bond involving the hydroxy O-atom and azomethine N-atom with graph-set notation S(6), as shown in Figure 2. Similar intramolecular hydrogen bonds are reported for the crystal structures of 2-(naphthalene-2-yliminomethyl) phenol and *N*-acetyl-4-[(2-hydroxybenzylidene)-amino]benzenesulfonamide monohydrate (Arunagiri *et al.*, 2013 (*a*); Chumakov *et al.*, 2005).

### S2. Experimental

An ethanol solution (25 ml) of chlorophenyl amine (0.25 mole) was mixed with hydroxy benzaldehyde (0.25 mole) and the contents were refluxed for 3 h and kept aside for crystallization. After a few days a pale yellow colour precipitate was formed. Recrystallization was from CHCl_3_/ethanol solution to form yellow needles. FT—IR (KBr pellet) in cm^-1^: 3437(O—H), 1614 cm^-1^(C=N stretching); ^1^H—NMR (400 MHz, DMSO– d^6^) in δ (p.p.m.) 13.17 (s,1*H*, aromatic O—H), 8.63 (s, 1H,*C*=N), 6.93 - 7.50 (m,8*H*, CH aromatic),^13^C—NMR (400 MHz, DMSO-d^6^) in δ (p.p.m.): 163.3 (C=N), 161.4 (phenolic OH), Electronic spectrum, λmax: 275 and 340 nm (due to intraligand π–π* and n–π* transitions); fluorescence spectra, 432 nm (attributed to the n–π* transition).

### S3. Refinement

All H atoms were positioned geometrically and treated as riding. The C—H and O—H bond lengths are 0.93 Å and 0.82 Å respectively.

### Crystal data

**Table:**

C_13_H_10_ClNO	*F*(000) = 480
*M**_r_* = 231.67	*D*_x_ = 1.360 Mg m^−^^3^
Orthorhombic, *P*2_1_2_1_2_1_	Mo *K*α radiation, λ = 0.71073 Å
Hall symbol: P 2ac 2ab	Cell parameters from 45 reflections
*a* = 6.8591 (2) Å	θ = 3.0–28.3°
*b* = 12.1829 (4) Å	µ = 0.31 mm^−^^1^
*c* = 13.5405 (5) Å	*T* = 293 K
*V* = 1131.50 (6) Å^3^	Cut needle, yellow
*Z* = 4	0.30 × 0.25 × 0.20 mm

### Data collection

**Table:**

Bruker Kappa APEXII CCD diffractometer	2315 reflections with *I* > 2σ(*I*)
Radiation source: fine-focus sealed tube	*R*_int_ = 0.017
Graphite monochromator	θ_max_ = 28.3°, θ_min_ = 3.0°
ω scans	*h* = −9→7
6509 measured reflections	*k* = −13→16
2744 independent reflections	*l* = −18→10

### Refinement

**Table:**

Refinement on *F*^2^	Secondary atom site location: difference Fourier map
Least-squares matrix: full	Hydrogen site location: inferred from neighbouring sites
*R*[*F*^2^ > 2σ(*F*^2^)] = 0.034	H-atom parameters constrained
*wR*(*F*^2^) = 0.097	*w* = 1/[σ^2^(*F*_o_^2^) + (0.0488*P*)^2^ + 0.0823*P*] where *P* = (*F*_o_^2^ + 2*F*_c_^2^)/3
*S* = 1.06	(Δ/σ)_max_ = 0.001
2744 reflections	Δρ_max_ = 0.17 e Å^−^^3^
146 parameters	Δρ_min_ = −0.17 e Å^−^^3^
0 restraints	Absolute structure: Flack (1983)
Primary atom site location: structure-invariant direct methods	Absolute structure parameter: 0.01 (7)

### Special details

**Table:**

Geometry. Bond distances, angles *etc*. have been calculated using the rounded fractional coordinates. All su's are estimated from the variances of the (full) variance-covariance matrix. The cell e.s.d.'s are taken into account in the estimation of distances, angles and torsion angles
Refinement. Refinement on *F*^2^ for ALL reflections except those flagged by the user for potential systematic errors. Weighted *R*-factors *wR* and all goodnesses of fit *S* are based on *F*^2^, conventional *R*-factors *R* are based on *F*, with *F* set to zero for negative *F*^2^. The observed criterion of *F*^2^ > σ(*F*^2^) is used only for calculating -*R*-factor-obs *etc*. and is not relevant to the choice of reflections for refinement. *R*-factors based on *F*^2^ are statistically about twice as large as those based on *F*, and *R*-factors based on ALL data will be even larger.

### Fractional atomic coordinates and isotropic or equivalent isotropic displacement parameters (Å^2^)

**Table:**

	*x*	*y*	*z*	*U*_iso_*/*U*_eq_	
Cl1	0.22873 (7)	0.87326 (5)	0.81891 (4)	0.0781 (2)	
O1	0.41345 (19)	0.60666 (15)	0.63294 (11)	0.0742 (5)	
N1	0.0830 (2)	0.68972 (13)	0.69278 (10)	0.0531 (4)	
C1	−0.0473 (2)	0.72426 (14)	0.76845 (12)	0.0486 (5)	
C2	0.0066 (2)	0.80739 (14)	0.83346 (13)	0.0513 (5)	
C3	−0.1161 (3)	0.84060 (16)	0.90860 (14)	0.0606 (6)	
C4	−0.2937 (3)	0.78949 (18)	0.92067 (14)	0.0672 (7)	
C5	−0.3476 (3)	0.70587 (18)	0.85879 (16)	0.0668 (7)	
C6	−0.2258 (3)	0.67361 (15)	0.78292 (13)	0.0563 (5)	
C7	0.0161 (2)	0.67467 (14)	0.60596 (13)	0.0506 (5)	
C8	0.1350 (2)	0.63031 (15)	0.52664 (12)	0.0501 (5)	
C9	0.3282 (3)	0.59651 (15)	0.54312 (14)	0.0556 (5)	
C10	0.4336 (3)	0.54992 (17)	0.46648 (17)	0.0676 (7)	
C11	0.3501 (3)	0.53771 (17)	0.37489 (16)	0.0716 (8)	
C12	0.1616 (3)	0.57133 (18)	0.35676 (15)	0.0701 (7)	
C13	0.0551 (3)	0.61655 (16)	0.43201 (13)	0.0597 (6)	
H1	0.33750	0.63660	0.67120	0.1110*	
H3	−0.07910	0.89710	0.95080	0.0730*	
H4	−0.37710	0.81180	0.97090	0.0810*	
H5	−0.46660	0.67070	0.86790	0.0800*	
H6	−0.26410	0.61710	0.74100	0.0680*	
H7	−0.11310	0.69270	0.59300	0.0610*	
H10	0.56110	0.52680	0.47710	0.0810*	
H11	0.42230	0.50620	0.32410	0.0860*	
H12	0.10740	0.56340	0.29420	0.0840*	
H13	−0.07260	0.63860	0.42020	0.0720*	

### Atomic displacement parameters (Å^2^)

**Table:**

	*U*^11^	*U*^22^	*U*^33^	*U*^12^	*U*^13^	*U*^23^
Cl1	0.0627 (3)	0.0739 (3)	0.0977 (4)	−0.0117 (2)	−0.0077 (2)	−0.0088 (3)
O1	0.0569 (7)	0.0894 (11)	0.0762 (9)	0.0072 (8)	−0.0098 (6)	−0.0086 (8)
N1	0.0541 (7)	0.0507 (8)	0.0545 (7)	0.0050 (6)	−0.0011 (6)	−0.0023 (6)
C1	0.0520 (9)	0.0442 (9)	0.0496 (8)	0.0093 (7)	−0.0039 (7)	0.0014 (7)
C2	0.0536 (8)	0.0423 (8)	0.0579 (9)	0.0038 (7)	−0.0103 (7)	0.0030 (7)
C3	0.0738 (12)	0.0493 (10)	0.0588 (10)	0.0110 (8)	−0.0099 (8)	−0.0079 (8)
C4	0.0715 (12)	0.0656 (12)	0.0644 (11)	0.0088 (10)	0.0122 (9)	−0.0050 (10)
C5	0.0633 (10)	0.0610 (12)	0.0761 (12)	−0.0017 (9)	0.0086 (9)	−0.0019 (10)
C6	0.0597 (9)	0.0477 (9)	0.0614 (9)	0.0011 (8)	−0.0033 (8)	−0.0063 (8)
C7	0.0516 (8)	0.0436 (8)	0.0566 (9)	0.0050 (7)	−0.0025 (7)	0.0009 (7)
C8	0.0578 (8)	0.0392 (8)	0.0533 (9)	−0.0004 (7)	0.0033 (7)	0.0024 (7)
C9	0.0547 (8)	0.0453 (9)	0.0667 (10)	−0.0036 (7)	0.0040 (8)	0.0034 (8)
C10	0.0609 (10)	0.0551 (11)	0.0869 (14)	0.0012 (9)	0.0190 (10)	0.0028 (10)
C11	0.0956 (15)	0.0490 (11)	0.0703 (13)	0.0000 (11)	0.0317 (11)	−0.0017 (9)
C12	0.0957 (14)	0.0612 (12)	0.0533 (10)	−0.0001 (12)	0.0053 (10)	−0.0001 (9)
C13	0.0702 (10)	0.0531 (10)	0.0559 (9)	0.0019 (9)	0.0001 (8)	0.0025 (8)

### Geometric parameters (Å, º)

**Table:**

Cl1—C2	1.7333 (15)	C9—C10	1.386 (3)
O1—C9	1.355 (2)	C10—C11	1.374 (3)
O1—H1	0.8200	C11—C12	1.378 (3)
N1—C7	1.275 (2)	C12—C13	1.369 (3)
N1—C1	1.423 (2)	C3—H3	0.9300
C1—C6	1.385 (2)	C4—H4	0.9300
C1—C2	1.392 (2)	C5—H5	0.9300
C2—C3	1.381 (3)	C6—H6	0.9300
C3—C4	1.378 (3)	C7—H7	0.9300
C4—C5	1.370 (3)	C10—H10	0.9300
C5—C6	1.381 (3)	C11—H11	0.9300
C7—C8	1.453 (2)	C12—H12	0.9300
C8—C13	1.404 (2)	C13—H13	0.9300
C8—C9	1.406 (2)		
			
C9—O1—H1	109.00	C11—C12—C13	119.17 (19)
C1—N1—C7	118.70 (13)	C8—C13—C12	121.26 (18)
N1—C1—C6	121.68 (15)	C2—C3—H3	120.00
C2—C1—C6	118.00 (15)	C4—C3—H3	120.00
N1—C1—C2	120.24 (13)	C3—C4—H4	120.00
Cl1—C2—C3	118.93 (14)	C5—C4—H4	120.00
C1—C2—C3	121.15 (15)	C4—C5—H5	120.00
Cl1—C2—C1	119.90 (12)	C6—C5—H5	120.00
C2—C3—C4	119.59 (17)	C1—C6—H6	120.00
C3—C4—C5	120.14 (19)	C5—C6—H6	120.00
C4—C5—C6	120.22 (19)	N1—C7—H7	119.00
C1—C6—C5	120.87 (17)	C8—C7—H7	119.00
N1—C7—C8	122.21 (13)	C9—C10—H10	120.00
C7—C8—C13	120.01 (14)	C11—C10—H10	120.00
C9—C8—C13	118.56 (16)	C10—C11—H11	119.00
C7—C8—C9	121.39 (15)	C12—C11—H11	119.00
O1—C9—C10	118.96 (18)	C11—C12—H12	120.00
C8—C9—C10	119.53 (18)	C13—C12—H12	120.00
O1—C9—C8	121.51 (17)	C8—C13—H13	119.00
C9—C10—C11	120.17 (19)	C12—C13—H13	119.00
C10—C11—C12	121.3 (2)		
			
C7—N1—C1—C2	−135.60 (17)	N1—C7—C8—C9	3.2 (3)
C7—N1—C1—C6	47.5 (2)	N1—C7—C8—C13	−179.23 (17)
C1—N1—C7—C8	−174.48 (16)	C7—C8—C9—O1	−1.9 (3)
N1—C1—C2—Cl1	2.9 (2)	C7—C8—C9—C10	177.01 (17)
C6—C1—C2—Cl1	179.83 (13)	C13—C8—C9—O1	−179.45 (18)
C6—C1—C2—C3	−1.7 (3)	C13—C8—C9—C10	−0.6 (3)
N1—C1—C2—C3	−178.70 (16)	C7—C8—C13—C12	−177.66 (18)
C2—C1—C6—C5	0.9 (3)	C9—C8—C13—C12	0.0 (3)
N1—C1—C6—C5	177.85 (17)	O1—C9—C10—C11	179.40 (19)
Cl1—C2—C3—C4	179.58 (15)	C8—C9—C10—C11	0.5 (3)
C1—C2—C3—C4	1.1 (3)	C9—C10—C11—C12	0.2 (3)
C2—C3—C4—C5	0.3 (3)	C10—C11—C12—C13	−0.8 (3)
C3—C4—C5—C6	−1.1 (3)	C11—C12—C13—C8	0.7 (3)
C4—C5—C6—C1	0.5 (3)		

### Hydrogen-bond geometry (Å, º)

**Table:**

*D*—H···*A*	*D*—H	H···*A*	*D*···*A*	*D*—H···*A*
O1—H1···N1	0.82	1.88	2.611 (2)	147
