# Supplementary figures and images for: Crystal structure of 2-{[(2-chloro­phen­yl)imino]­meth­yl}phenol
Source: Acta Crystallogr E Crystallogr Commun. 2015 Jan 1;71(Pt 1):o48. doi: 10.1107/S2056989014026978 (PMC4331896; doi:10.1107/S2056989014026978)

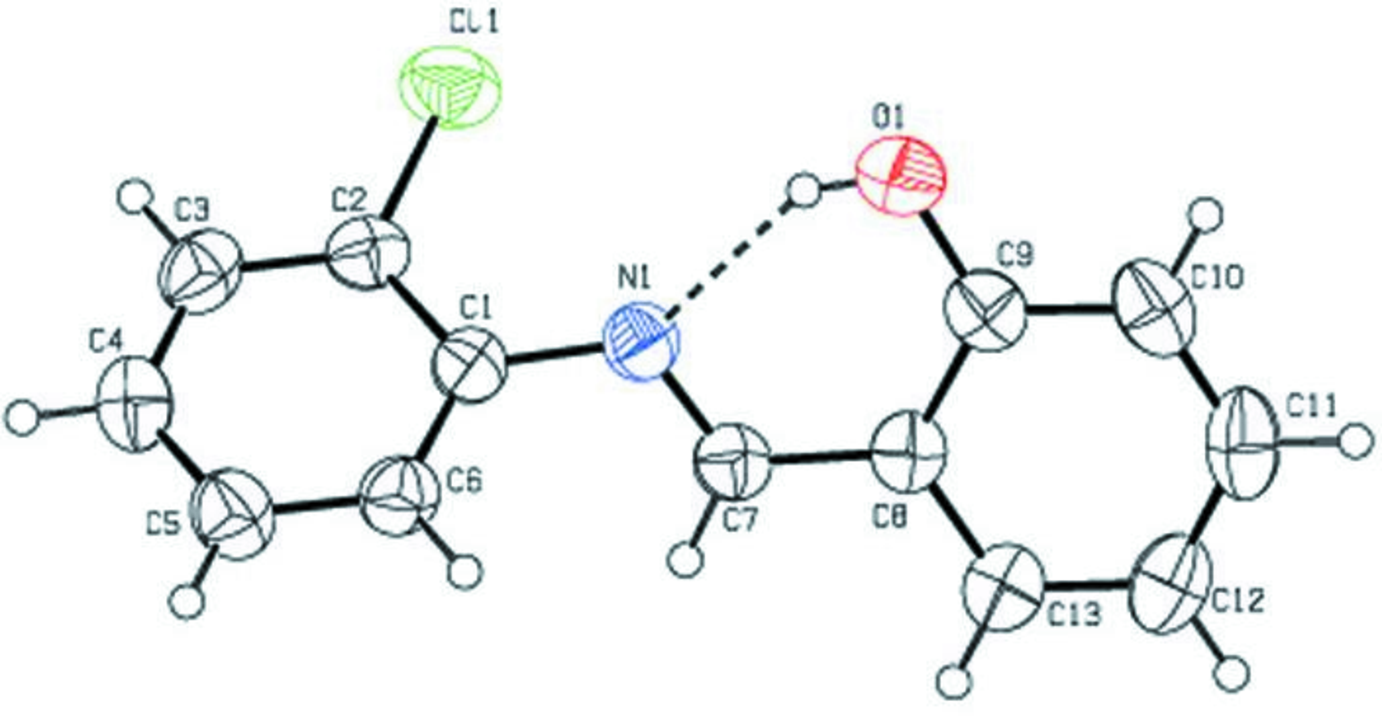

Supplement: Supplementary file 4 [file e-71-00o48-fig1.tif]

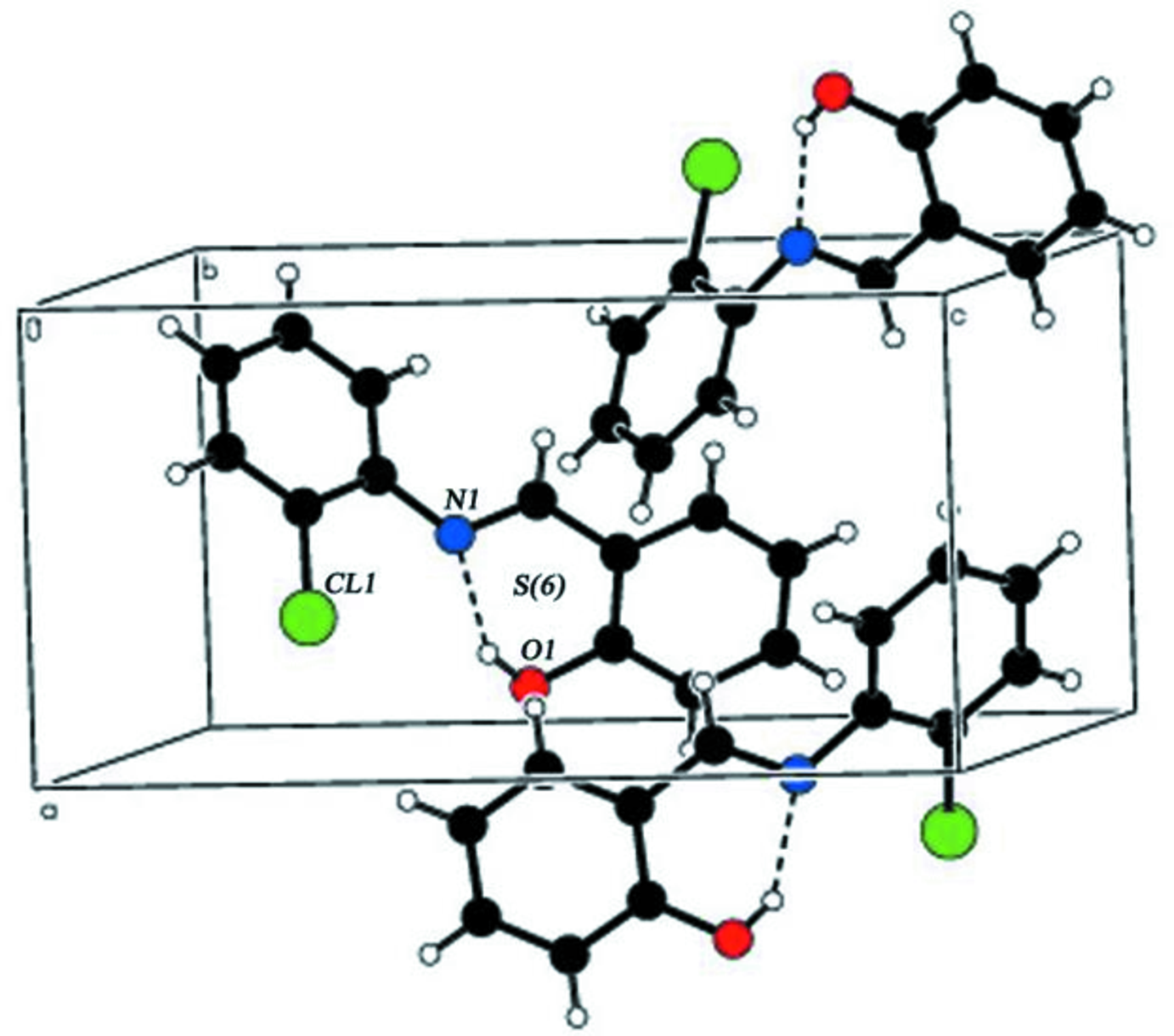

Supplement: Supplementary file 5 [file e-71-00o48-fig2.tif]
